# Integrated working in local authority decision-making about air quality: a qualitative study in Southwest England

**DOI:** 10.1093/pubmed/fdad036

**Published:** 2023-05-05

**Authors:** Andrew Turner, Adele Webb, Russ Jago, Sara Blackmore, Frank de Vocht, Jeremy Horwood

**Affiliations:** National Institute for Health Research Applied Research Collaboration West (NIHR ARC West), University Hospitals Bristol and Weston NHS Foundation Trust, Bristol BS1 2NT, UK; Centre for Academic Primary Care, University of Bristol, Bristol BS8 2PS, UK; National Institute for Health Research Applied Research Collaboration West (NIHR ARC West), University Hospitals Bristol and Weston NHS Foundation Trust, Bristol BS1 2NT, UK; National Institute for Health Research Applied Research Collaboration West (NIHR ARC West), University Hospitals Bristol and Weston NHS Foundation Trust, Bristol BS1 2NT, UK; Centre for Exercise, Nutrition & Health Sciences University of Bristol, Bristol BS8 1TZ, UK; Population Health Sciences, Bristol Medical School, University of Bristol, Bristol BS8 2PS, UK; Population Health Sciences, Bristol Medical School, University of Bristol, Bristol BS8 2PS, UK; Department of Health and Social Care, Office for Health Improvement and Disparities, 39 Victoria Street, London SW1H 0EU, UK; National Institute for Health Research Applied Research Collaboration West (NIHR ARC West), University Hospitals Bristol and Weston NHS Foundation Trust, Bristol BS1 2NT, UK; Population Health Sciences, Bristol Medical School, University of Bristol, Bristol BS8 2PS, UK; National Institute for Health Research Applied Research Collaboration West (NIHR ARC West), University Hospitals Bristol and Weston NHS Foundation Trust, Bristol BS1 2NT, UK; Centre for Academic Primary Care, University of Bristol, Bristol BS8 2PS, UK

**Keywords:** air quality, decision-making, qualitative, local authorities

## Abstract

**Background:**

Exposure to poor air quality is one of the most significant environmental public health challenges. In the UK, local authorities (LAs) are responsible for monitoring and managing air quality. This article explores the need and mechanisms for cross-departmental working in LAs to make decisions about air quality issues.

**Methods:**

Semi-structured qualitative interviews with public health, environmental health and transport staff from LAs within the Southwest of UK. Interviews were conducted between April and August 2021 and analysed using a thematic approach.

**Results:**

In sum, 24 staff from 7 LAs participated. Local authority staff in public health, environmental health and transport teams recognized that managing air quality was a cross-departmental issue. To enable effective integrated working staff described four successful mechanisms: (i) policy commitments and political support; (ii) dedicated air quality steering groups; (iii) existing governance and oversight groups; and (iv) networking and relationships.

**Conclusions:**

This study has identified mechanisms that LA staff have found support cross-departmental and integrated working on air quality issues. These are mechanisms that have helped environmental health staff work towards achieving compliance with pollution limits, and that have helped public health staff get air quality considerations recognized as a wider health issue.

## Introduction

Exposure to poor air quality is one of the most significant environmental public health challenges.^1–4^ The World Health Organization (WHO) estimate that annually there are around 4 million deaths related to air pollution, globally.^2^ In the UK, it has been suggested that poor air quality reduces life expectancy and may cause around 30 000 premature deaths annually.^5^^,^^6^

Pollution from transport is one of the biggest influences on air quality and makes up around 80% of nitrogen dioxide emissions in areas where the UK is exceeding NO2 limits.^4^ Transport systems have impacts on air quality through their effects on congestion and the travel choices they promote. Measures available to tackle air pollution from traffic include reconfiguring transport systems, creating clean air zones,^4^ and introducing measures to promote walking and cycling (also known as ‘active travel’). Changes that improve air quality and increase active travel (and therefore how much physical activity people do) are likely to improve people’s health.^6–9^ In the UK, it has been noted that deaths due to poor air quality constituted a public health emergency.^10^

In the UK, local authorities (LAs) are responsible for monitoring and managing air quality.^11^^,^^12^ The Environment Act 1995 sets the framework for local air quality management which local authority environmental health teams work within, reporting annually to the Department for Environment, Food and Rural Affairs (DEFRA).^13^ Where air quality objectives are not being met, and if there is a relevant exposure to the public, an Air Quality Management Area (AQMA) must be declared and an Air Quality Action Plan (AQAP) produced for improving air quality in the area.^12^ The vast majority of AQMAs have been declared because of an exceedance in levels of nitrogen dioxide.^13^ Recently, some LAs in the UK have also begun implementing Clean Air Plans (as instructed by central government) that create ‘clean air zones’ in which certain types of vehicle are charged to enter city centre areas.^4^

Previous research has found that AQAPs have had limited effectiveness in improving air quality because the sources of poor air quality are difficult for LAs to control^14^^,^^15^ (e.g, ‘growth in road traffic, commerce and industry’^14^) and because implementing AQAPs requires close links between environmental health and transport teams^16–19^ that may not be well-developed or prioritized.^19^ These problems are despite existing policy guidance,^12^ as well as National Institute for Health and Care Excellence (NICE) publishing recommendations for strategies to improve air quality and health aimed at local authority staff and elected members.^20^ Also, although public health teams have the expertise and the evidence^21^ to contribute to understanding the factors affecting air quality and the impact on health, and their involvement is included in policy guidance,^12^ the role of public health in decision-making around air quality issues has been found to be under developed.^22–24^

Air quality is a cross-departmental issue for LAs.^25–28^ Environmental health teams are responsible for regulatory compliance, but changes implemented by transport teams often have the potential for the biggest impacts on air quality. Furthermore, public health teams have a key interest in air quality both in terms of the health effects of exposure to air pollution and the causes of air pollution (such as congestion) that are themselves closely linked to other public health issues such as obesity and physical activity.

This article explores the need and mechanisms for cross-departmental working to make decisions about air quality, in seven LAs in the Southwest.

## Methods

### Sampling and recruitment

Seven LAs within the Southwest of UK participated in the study. These LAs have a mix of populations, sizes and cover a range of urban, semi-urban and rural areas. Six are unitary authorities and one is a two-tier (county and district) authority.

Staff within the LA’s public health, environmental health, and transport teams were identified from publicly available documents (for example, Annual Air Quality Status Reports) or from existing contacts. Selected staff were emailed study information and invited to take part in an interview. Snowball sampling was used to identify further individuals with relevant experience of air quality issues.

Participant sampling and recruitment, data collection, and analysis were conducted in parallel to allow for the continuous assessment of the suitability and ‘information power’ of the sample with regard to study aims.^29^

### Data collection

Semi-structured interviews were conducted by AT, using a flexible topic guide informed by existing literature to explore what informs local authority decision-making around air quality and transport plans. The topic guide was refined iteratively as interviews and preliminary analysis progressed. (The final iteration is available as Supplementary File 1.)

Interviews were conducted between April and August 2021 by telephone or video-call and audio recorded. All participants provided informed consent and interviews lasted between 30 and 60 minutes. Recordings were transcribed verbatim.

### Analysis

Interviews were fully transcribed and coded using QSR NVivo 12 software and analysed using an inductive thematic approach.^30^ A combination of deductive coding, based on the aims of the study and the topic guide, and inductive coding, identifying themes within the data, was used. Line-by-line coding was used to construct draft coding frames. Three transcripts were coded independently (AT JH) and discrepancies discussed, to aid the generation and refinement of codes and to maximize rigour. AT then applied the refined coding frame to all the transcripts, with regular meetings with JH to discuss emerging findings. Finally, AT used charting to identify patterns in the data and drafted a narrative based on the analysis.

## Results

The number of LAs and participants involved are shown in Table 1. For each subheading of the results, illustrative quotations from the interviews are shown in Table 2.

**Table 1:** Participants within each local authority

**Local authority**	**Local authority staff interviewed by department**			
	Environmental health	Public health	Transport	**Total**
LA1		1	2	**3**
LA2	2	2	1	**5**
LA3	1	1	2	**4**
LA4	2	1		**3**
LA5	1		1	**2**
LA6	1	1	2	**4**
LA7	1	2		**3**
**Total**	**8**	**8**	**8**	**24**

**Table 2:** Illustrative quotes from interviews

**Integration and cross-departmental working**	
**Need for integration**	
	‘the principle problem and the one that everyone has to fix is that many different departments or interests and ends have to work together to fix the air quality problem.’ Environmental Health, LA5
	‘the vast majority of the time [we’re] reliant on getting funding from other areas […] Action plans in the past, I think most people would agree, it’s all very well saying “Here’s a problem with air pollution and here’s what we think you should do with it”, but that whole regime doesn’t come with any resource. It’s all monitoring and red lines on a map, but how do you actually get things done? […] if you can work together and utilise the traditionally much larger transport budgets then, yes, you’re onto something.’ Environmental Health, LA2
	‘[Transport’s] funding comes from central government, the transport interventions, and often that’s very, very targeted. […] you get a very bespoke funding to deliver certain types of cycle projects or projects that deliver certain benefits around productivity. But what it’s not is the council transport team sat there like Smaug on a big heap of gold deciding exactly how it’s gonna fund and do everything. We don’t quite have that control over our funding […] it comes with very specific and strict conditions.’ Transport, LA3
	‘it’s usually much better if we can do something that benefits air quality but which also is dealing with other factors [… to] hit a number of objectives: air quality or traffic management, even demand management—but typically we don’t do things purely for air quality benefit.’ Transport, LA6
**Regulatory compliance**	
	‘[Transport’s] overarching target is the compliance one. So you know bringing all those monitoring sites down […] transport being the biggest contributor to nitrogen dioxide in particular, […] Achieving compliance across all of those sites in the shortest time possible and that’s our statutory—that’s what required of us as a statutory requirement.’ Transport, LA3
	‘when you’re developing an action plan, that brings everyone together. And that action plan gives you a list of targets that everyone is responsible for, so that holds people to account, it makes people own that measure. So if the measure is a traffic light system in […the town], then Transport and Highways own that and you have meetings based on progress towards that target and it may take a while, it might not be something that happens straightaway, as long as everyone’s clear. So I think the Air Quality Action Plan is the thing that brings everyone together.’ Environmental Health, LA6
**Wider health impacts of air quality**	
	‘Public Health felt that as a council it [air quality] was quite disjointed and very much based on the regulatory side of things and there was opportunity for Public Health to influence there to maybe widen things out to say we’re not just concentrating on the two air quality management areas and we need to have a bigger perspective on air quality […]we have this bigger ambition that we don’t want to just talk about traffic lights.’ Public Health, LA1
	‘In normal times, the role would very much be around technical advice in terms of the public health aspect and liaison between Public Health England who have the real kind of techy people, about air quality and the science behind it and then the air quality agenda’ Public Health, LA7
**Mechanisms that facilitated more integrated working**	
**Steering groups**	
	‘There is a working group on air quality which includes transport, public health and environmental health and then that feeds into climate change work as well’ Environmental Health, LA3
	‘I had discussions with air quality team and environmental health and we said “look, you need to get a steering group across the council, in effect, because there’s too many players involved,” so they set up a steering group. […] I needed to meet with transport planners, the designers of housing estates, the traffic teams, the cycling teams, and what was quite apparent was the links weren’t there, certainly with public health, and the way we see it [public health needs to be in] policy across the council [… The steering group is] led by public protection but when it’s got a steer and emphasis from public health centrally across the council then it makes that a little bit easier.’ Public Health, LA6
	‘The steering group just makes a bit of noise and speaks to the right people and just shifts things forward. […] the modes of action are quite subtle, but that’s about having [e.g.] the head of planning in the air quality steering group, so he knows what priority the air quality is and he can tinker with bits and bobs in his own group. […] everyone knows the air quality steering group reports to the chief exec and it’s for something that’s considered a big problem: an air quality exceedance in the town. So we have that kind of veiled power, but most of the power just comes from getting the people in the room and getting them talking about the same subject.’ Environmental Health, LA5
	‘[I] sit on the health protection assurance group. Annually I will just feed into that, in terms of the air quality annual status reports. So I just give that group an update on where we are in terms of air quality. So […] they’re aware of what’s going on, and if anything needs to be implemented, they can make that happen.’ Environmental Health, LA4
	‘As officers, we do engage with our public health colleagues and also the air quality [team …] we have a regular meeting, but there’s not really something there at steering group level, and the closest I suppose we get is when it goes into the political member arena where the environment select committee.’ Transport, LA6
	‘we’ve got the various boards, and there’s been a lot of work over the last two years on the boards working together. So that gives a lot of room for cross-fertilisation. So just to give you an example, the health and wellbeing board had a fantastic development session with the environment board and you know that’s really helpful in terms of thinking about priorities for the coming year.’ Public Health, LA3
**Policy commitments and political support**	
	‘[LA6] has an air quality strategy, it was implemented in 2019 so that is very helpful for us because it’s a high-level document, it’s adopted by members and so it has a democratic clout to it as well as a sort of Officer sort of, yeah, so it does act as almost a way of us being able to you know, obviously there are statutory functions but you know when it comes to planning applications, when it comes to you know, providing incentives, when we’re looking at other aspects that maybe aren’t statutory but we know are going to improve, have a big impact, it allows us to refer to that document and it carries weight in decision-making. So it does help sort of fly the air quality flag at the highest level when you’ve got a document like that.’ Environmental Health, LA6
	‘it’s a little bit frustrating that we don’t have an air quality strategy or an action plan which we can explicitly as a team draw upon and say, “This is our action plan. It’s been approved and we support these kind of initiatives because of air quality.” […] it’s just always useful to have those documents which have been approved and adopted by councils and approved by full council, and say “Look, you said that this is a priority.”’ Environmental Health, LA3
**Networking and relationships**	
	‘you need a Public Health person sitting in every planning team, which some local authorities have done. They’ve seconded a Public Health person or pay for someone in planning to say, “What are the health impacts of this?”’ Public Health, LA7
	‘Basically my role is to integrate public health into most policies so I actually have a counterpart in the planning team, one of the senior planners that I meet and I’ve managed to bring along to Southwest regional meetings in public health […] there are resources being given because I’m allowed to take a planning officer to a regional meeting. That’s quite a commitment for planning to do that. So, it’s more people resource and time that I’m allowed to go and meet [senior staff…] to really outline what my idea is for integration, which has been good.’ Public Health, LA6
	‘It’s that investment to talk about it with other departments and raise the profile with elected members and all those sorts of things. That’s why you need bodies in posts to do that […] I’m the resource. I don’t have a budget and I don’t have a team. I don’t have any enforcement roles with other council departments so it is an educative influencing sort of role and trying to align things with the overall council objectives. It’s speaking to other departments and saying if you do this then it helps you align with the council. It’s that sort of influencing role.’ Public Health, LA1
	‘public health really was an outsider to these teams and the pandemic’s certainly broken down those barriers […] I’m keen to make sure we continue with these relationships now and lucky enough I had the chance to talk to the director of the travel and planning teams and then talked about the [COVID-19] steering group and said ‘we need public health to be sat on that” and that’s developed. […] the good thing of the pandemic is it’s opened doors which were never easy to open, if I’m honest with you.’ Public health, LA6

### Integration and cross-departmental working

#### Need for integration

The need for integrated and cross-departmental working was recognized by environmental health, public health and transport staff as being necessary to address air quality issues. Air quality issues included questions about what measures to put in place to reduce levels of a particular pollutant in specific places (e.g. a single road junction), through to more general questions about the impact on air quality, of activities such as housing developments or larger-scale changes to transport systems designed to reduce congestion.

For environmental health staff, the regulatory framework around air quality (the ‘Local Air Quality Management Regime’) made it clear how air quality should be monitored and reported, and how action plans should be developed to address exceedances of pollutants. However, designing and implementing AQAPs required collaboration and buy-in from colleagues in other teams who had responsibility for making the recommended changes; mostly transport and planning teams who were responsible for road changes.

Collaboration between environmental health and transport teams was argued to be critical because environmental health teams also typically did not have their own funding to implement action plans. Implementation was therefore conditional on being able to make air quality improvement measures in the actions plans a complementary part of other projects or bids, rather than through projects that benefited air quality alone. Equally other complementary projects and bids may be constrained in how their resources could be used, which, in turn, determined the scope of integration between teams.

#### Regulatory compliance

The context for integration between environmental health and transport teams was largely focused around implementing measures to achieve compliance with the statutory aspects of air quality management and ensuring compliance with legal thresholds for air quality. For example, working together to implement measures in AQAPs that were concerned with improving air quality in AQMAs, or as part of larger government-directed Clean Air Plans. Examples included targeted measures such as Traffic Regulation Orders to prevent HGVs travelling along a small length of road, through to larger projects such as the implementation of Clean Air Zones in city centre areas.

AQAPs could function as the catalyst for cross-department working, because it was necessary for environmental health officers to consult colleagues in other departments to agree the action plan. Conversely, without an action plan, it was considered more challenging for environmental health officers to collaborate with other departments, which, in turn, made integration more difficult before an AQMA was established.

#### Wider health impacts of air quality

The wider issues of air quality beyond regulatory compliance were mostly considered by public health staff and less commonly considered by other teams. Public health staff emphasized the need for integration with transport and planning teams for embedding a holistic consideration of health, which included the health effects of poor air quality as well as factors that affect air quality.

The air quality work of public health teams was focused on getting air quality and health considerations onto the agenda of other departments’ decision-making. For example, integration and cross-department working often meant public health staff fulfilling the role of translating evidence and technical advice (e.g. from universities, UK Health Security Agency, or internal sources) to support other teams. Or equally, advocating that impacts on air quality (and impacts of poor air quality) are an important health issue.

Public health staff noted that they typically didn’t lead air quality work and instead their role tended to involve both advocating for how health should be considered (although they may lead on health promotion projects), as well as providing more specific input on the impacts on air quality of interventions being designed and implemented by other teams. For example, public health staff described contributions to interventions such as the closing of streets to traffic around schools (because this would be more naturally led by the teams that could put in place the necessary traffic orders), or lending support to plans for active travel infrastructure that had been designed by transport teams. In these cases, the projects led by other teams were not typically air quality projects, but projects with other aims that potentially affected the air quality, such as the creation of new cycle paths.

### Mechanisms that facilitated more integrated working

#### Policy commitments and political support

Local and national policies, local strategy documents, and high-level policy commitments were all highlighted as important for supporting the recognition of air quality issues across LAs. National policies helped establish the importance of air quality issues, for example though criteria for funding bids that required consideration of air quality. Local policies provided tangible examples of LAs’ priorities and commitments, which environmental health staff could use to support the importance of considering air quality in decision-making processes. Conversely, making these arguments was described as being difficult without a concrete strategy or policy commitment to refer to.

#### Steering groups

Some LAs had established steering or working groups that provided a formal mechanism for bringing together representatives from different teams to address air quality issues. Inclusion of transport, public health and environmental health staff was most common, but occasionally climate change, planning and staff from other teams also took part, depending on the scope of the issues that a steering group addressed.

Air quality steering groups are an established mechanism in LAQM policy for implementing action plans and ensuring regulatory compliance. They provided a forum for discussing the content of Annual Air Quality Status Reports (a statutory document submitted to DEFRA each year by environmental health teams in each LA in the UK) and the progress made towards previous actions. Regulatory compliance was not the only aspect of air quality that some steering groups considered, and not necessarily the reason why groups had been established, however. In some cases, steering groups could be a powerful mechanism for bringing people together and agreeing actions on wider air quality issues, when there was high-level support, particularly if steering groups also included elected members.

#### Other existing governance and oversight groups

Existing governance and oversight groups (separate from dedicated air quality steering groups) provided an avenue for facilitating cross-departmental working on air quality, although these were more limited in their engagement with air quality issues than dedicated steering groups.

For example, health protection groups could provide a way for environmental health staff to collaborate with public health staff when those groups reviewed Annual Air Quality Status Reports prior to submission to DEFRA. The planning and development process could also provide a way for environmental health staff to interact with transport-led projects. For example, through being consultees and commenting on the air quality impact of proposals to create new roads or developments. Political committees provided another forum where air quality issues could be addressed cross-departmentally. AQAPs were sometimes reviewed at Environment Select Committees or similar committees. Cross-departmental working was facilitated further by various boards within LAs, such as health and wellbeing boards, and environment boards, whose responsibilities and priorities overlapped with air quality issues.

#### Networking and relationships

In some cases, there were formal roles for public health staff to be involved in other departments’ meetings, or even to be seconded to those departments. Equally, some departments committed time for their staff to work with public health staff. For example, attending public health meetings to learn more about the ‘language’ and ‘principles’ of public health. More commonly however, cross-departmental working was established informally. Opportunities to build and maintain relationships between staff was seen as key, such as ad hoc opportunities to attend meetings or give presentations to other departments, which themselves were created by existing personal relationships. For public health staff, such opportunities were created when staff had dedicated time to network, educate and build relationships with other departments. Having this dedicated time was highlighted as critical to influencing other departments’ decision-making around air quality and its health impacts.

Relationships were also built through prior collaborative work. Larger scale projects where environmental health, public health and transport teams had worked together were sometimes credited with establishing relationships between teams that continued when smaller more ad hoc projects were being conducted. Some public health staff noted that relationships formed as a result of new ways of working in response to the COVID-19 pandemic were expected to have beneficial consequences for the future.

## Discussion

### Main finding of this study

Local authority staff in public health, environmental health and transport teams recognized that managing air quality was a cross-departmental issue. Environmental health teams conducted monitoring and action planning, but transport was often best placed to implement measures. Integrated working between these teams was typically focused on achieving regulatory compliance. In contrast, the role played by public health teams involved widening the scope of air quality issues beyond compliance and thinking more holistically about health. Resources and funding to address air quality issues were often ad hoc and relied on finding synergies with funding bids for other purposes, limiting the staff’s ability to be strategic and address local problems holistically.

**Fig. 1:**
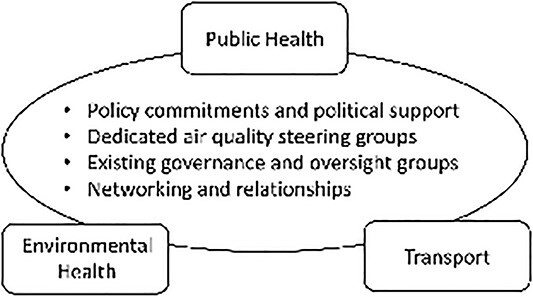
Mechanisms that facilitated integrated working on air quality issues.

To enable effective integrated working staff described a series of successful mechanisms, namely (i) national and local policy documents were useful to support staffs’ ability to make the case for consideration of air quality issues in decision-making by providing tangible evidence of LA commitments to improving air quality; (ii) dedicated air quality steering groups, which brought together key people from different teams to address air quality issues; (iii) using existing governance and oversight mechanisms to raise air quality issues, for example, using forums such as health protection groups, health and wellbeing boards and political committees; and (iv) the ability to build and maintain relationships with other teams– in terms of having dedicated time and, or, a formal role within other teams—was highlighted as critical to influencing other department’s decision-making around air quality and its health impacts. These are illustrated in Figure 1.

### What is already known on this topic

Integration between environmental health and transport teams in LAs has been found to be a barrier to implementing AQAPs.^16–19^ Equally, air quality has been found to be a low priority in local authority decision-making processes.^19^ Furthermore, previous studies have argued that the role of public health in decision-making around air quality issues is under developed.^22–24^

Beattie et al. investigated the integration of air quality management in local authority decision-making in 2004.^26^ Although this was conducted prior to LAs being given responsibility for public health, Beattie et al. similarly found that dedicated air quality groups could be an important mechanism for integrated and cross-departmental working.

Le Gouais et al. examined local authority decision-making for active living infrastructure (ALI) finding that public health practitioners with influence, resources and working within supportive policy environments were able to make health part of the decision-making agenda when ALI was designed and built.^31^ These three key factors are all present in the mechanisms identified in this study for facilitating integration between teams on air quality issues and alongside those specific mechanisms constitute more general ways in which the role of public health could be developed, namely increasing influence, resources and supportive policies.

### What this study adds

This study adds the perspective of public health teams to a topic that has often been researched in relation to integration between environmental health and transport teams. This study also uses qualitative interviews with local authority staff to provide in-depth understanding and expand on similar studies that have looked at integrated working using surveys, documentary analysis and case study methods.

This study has identified mechanisms that environmental health, public health and transport staff have found support cross-departmental and integrated working on air quality issues. These are mechanisms that have helped environmental health staff implement AQAPs aiming at achieving compliance with pollution limits, and that have helped public health staff get air quality considerations recognized as a wider health issue.

### Limitations of this study

This study involved LAs in the Southwest. The Southwest has a mix of urban, semi-urban and rural authorities, however air quality issues in the region may not be nationally representative. Furthermore the majority of LAs involved in this study are unitary authorities. The additional separation between departments in two-tier authorities may raise additional issues not captured in this study.

This study only interviewed local authority staff in environmental health, public health and transport teams. As air quality is a cross-cutting issue, the views of planning teams, and other relevant teams, could also be considered in future research. The views of elected officials are also important to further understand the political context within which local authority staff work and the influence of national and local party policy.

The study took place from April–August 2021 when public health departments in LAs were still heavily focused on responding to the COVID-19 pandemic and when their capacity to engage in air quality work had been reduced since the start of the pandemic in March 2020. Participants were often reflecting on pre-pandemic experiences, or on roles that they currently did not have the capacity to perform.

## Supplementary Material

Clearways_LA_AQ_decision_making_TOPIC_GUIDE_suppl_file_1_v1_fdad036

## Data Availability

The data underlying this article are available in the data.bris Research Data Repository at https://data.bris.ac.uk, and can be accessed through a data access request.

## Notes

**Andrew Turner**, Senior Research Associate

**Adele Webb**, Public contributor

**Russ Jago**, Professor of Physical Activity & Public Health

**Sara Blackmore**, Deputy Director South West Population Health & Wellbeing

**Frank de Vocht**, Professor in Epidemiology and Public Health

**Jeremy Horwood**, Professor of Social Science and Health
