# Supplementary material for: Integrated working in local authority decision-making about air quality: a qualitative study in Southwest England
Source: J Public Health (Oxf). 2023 May 5;45(3):654–62. doi: 10.1093/pubmed/fdad036 (PMC10470340; doi:10.1093/pubmed/fdad036)
Supplement: Clearways_LA_AQ_decision_making_TOPIC_GUIDE_suppl_file_1_v1_fdad036 [file clearways_la_aq_decision_making_topic_guide_suppl_file_1_v1_fdad036.docx]

Supplementary File 1:

Local Authority Staff Interview Topic Guide


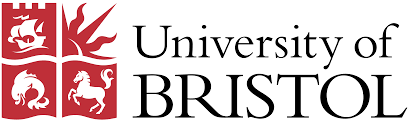


CLEARWAYS Study

Examining air pollution and active travel

**Local Authority Staff Interview Topic Guide**

**Introduction and verbal consent**

- Introduce self
- Re-cap aim of study
- Re-cap consent: Explain voluntary participation, audio record, anonymous quotes
- Switch audio recorder on
- For the audio recording, can I check that:
  - You read and understood the study information sheet?
  - You know that taking part in the interview is voluntary and you are free to stop the interview at any point?
  - You agree to our conversation being audio recorded?
  - You understand that quotations from the interview may be used to illustrate our findings, but it will not be possible to trace who said them?
  - You understand that your comments cannot be removed once the transcription has been anonymised.
  - You understand that any personal and identifiable information you give will be kept confidential unless something you say indicates that you or someone else is at risk of harm.
  - You understand that in the future an anonymised transcript of your interview may be shared with other researchers for use in other studies. All such requests for sharing will be reviewed by the University of Bristol Research Data Access Committee.
  - You agree to take part in the study?

**Start**

- Can you briefly describe your role in the local authority?
- Can you briefly describe the local air quality strategy?
- How does transport strategy fit into this picture?
- How does active travel fit into this picture?

**Required AQ monitoring and management**

Thinking about the statutory air quality monitoring and management you do...

AQMAs & Action Plans

- [If they have any AQMAs…] can you briefly tell me a little about your AQMAs?
- What have you done in these areas to improve air quality? (Action Plans)
- What informs the design and implementation of your AQ Action Plans?
  - Resources
  - Trade-offs / competing priorities (transport, public health, local economy etc)
- What interventions were available / considered?
- How have you consulted with the public and other organisations (e.g. local businesses, places of worship, schools)?
  - What is the aim of the consultation?
  - How does it influence what gets put in place?

AQ Data

- What air quality data is routinely collected?
  - Format and availability of this data?
- How good is the data/modelling for informing your AQ management?
  - How do you use the data?
  - What could be better?
  - What else would help?
- [If not AQ specialist] How do you work with the AQ team?

**Wider use of AQ data**

Apart from the statutory requirements to monitor and manage AQ...

- How is AQ work used to inform the local authorities strategies and plans? - e.g. public health, transport works, other departments/teams?
- To what extent do air quality considerations influence transport policy/plans? - Formally or informally
- What affects your ability to influence / make changes to those plans?
- What decisions have been made in recent years, where air quality considerations played a role? - What schemes/interventions have been put in place? – WHY?
- What behaviour changes and health impacts are anticipated? – How is this evaluated?
- How good is your air quality data/modelling for informing transport plans? - What could be better?
- What evidence would have been helpful to make decisions?
- What else would have been helpful to make decisions?
- What would help future decision making?

**Changes in response to COVID-19**

- How have air quality management plans changed because of the COVID-19 pandemic?
- How have transport and / or active travel plans changed because of the COVID-19 pandemic?
- How do these changes fit into your previous transport and air quality strategies?
  - Have the changes in (public) behaviour due to COVID-19 facilitated a more rapid implementation of air quality management plans, if so how?
- How did you consult with the public and other organisations?
- What are your future plans for these changes?
- What feedback have you had on recent changes?
- What impacts have the recent changes had?

**Public Health specific questions**

- How does public health use air quality data?
- To what extent is air quality treated as a public health issue?
- How do air quality issues inform public health priorities and strategy?
- How does public health integrate/influence other departments around air quality?
  - What affects your ability to integrate/influence?
  - What mechanisms are there to enable it?
  - What would help?

**Finish**

- What do you think you do well, that it would be helpful to share with other local authorities?
- What other key aspects of air quality management have I not asked you about?
